# Presepsin Levels in Pediatric Patients with Fever and Suspected Sepsis: A Pilot Study in an Emergency Department

**DOI:** 10.3390/children11050594

**Published:** 2024-05-15

**Authors:** Antonio Gatto, Lucia Mantani, Caterina Gola, Valeria Pansini, Lorenzo Di Sarno, Lavinia Capossela, Serena Ferretti, Benedetta Graglia, Antonio Chiaretti

**Affiliations:** 1 Department of Pediatrics, Fondazione Policlinico Universitario A. Gemelli—IRCCS, 00168 Rome, Italy; valeria.pansini@policlinicogemelli.it; 2 Department of Pediatrics, Università Cattolica del Sacro Cuore, 00168 Rome, Italy; lucia.mantani01@icatt.it (L.M.); caterina.gola01@icatt.it (C.G.); lorenzo.disarno01@icatt.it (L.D.S.); lavinia.capossela01@icatt.it (L.C.); serena.ferretti01@icatt.it (S.F.); benedetta.graglia01@icatt.it (B.G.); 3 Department of Women’s Health Sciences, Fondazione Policlinico Universitario A. Gemelli—IRCCS, 00168 Rome, Italy; antonio.chiaretti@policlinicogemelli.it

**Keywords:** presepsin, biomarker, sepsis, pediatric emergency department, infection

## Abstract

Sepsis is a life-threatening condition that affects 1.2 million children annually. Although there are several criteria for diagnosing this condition, signs are often nonspecific, and identifying sepsis is challenging. In this context, presepsin (P-SEP) seems to be a promising new biomarker since its plasma levels increase earlier than other sepsis-related proteins and its measurement is faster. We enrolled 157 minors who presented to the Pediatric Emergency Department of Agostino Gemelli Hospital with fever and suspected sepsis. Biochemical, anamnestic, and clinical data were collected. Viral agents were identified as the causative factor in 64 patients, who had an average P-SEP value of 309.04 pg/mL (SD ± 273.2), versus an average P-SEP value of 526.09 pg/mL (SD ± 657) found in 27 bacterial cases (*p* value: 0.0398). Four cases of overt sepsis had an average P-SEP value of 3328.5 pg/mL (SD ± 1586.6). The difference in P-SEP levels in viral versus bacterial infections was found to be statistically significant; therefore, P-SEP may have a central role in the evaluation of febrile children, helping clinicians distinguish between these two etiologies. Furthermore, amongst the cases of confirmed sepsis, P-SEP was always greater than 2000 pg/mL, while C-reactive protein and procalcitonin values appeared lower than what was considered significant.

## 1. Introduction

Pediatric sepsis is a life-threatening condition arising from an uncontrolled host response to confirmed or suspected infection and is characterized by rapid and potentially fatal progression [1]. The global incidence of sepsis among children is estimated at 1.2 million cases annually, and the condition has a mortality rate that remains elevated (up to 25% in certain settings) [2].

Defining sepsis proves challenging, as evidenced by numerous consensus definitions published by international task forces over the years. The distinctive compensatory reserve of the pediatric population further complicates the clinical picture, as the initial signs and symptoms of sepsis in children can be subtle. Distinguishing a potentially septic child from one affected by viral illness can pose a significant diagnostic challenge [3,4].

From a microbiological standpoint, the definitive confirmation of an underlying infection involves a positive culture, which remains the gold standard for the identification of microorganisms. However, latency of results and mediocre accuracy greatly diminish its utility in the initial management of sepsis, especially in emergency settings [5].

Biochemical markers of inflammation, such as C-reactive protein (CRP), procalcitonin (PCT), and interleukin-6, have been reported as potential indicators of systemic inflammatory response syndrome (SIRS); however, none of these indexes are robust enough to be recognized as actual diagnostic criteria [1]. In current clinical practice, CRP and PCT are the most frequently used biomarkers, representing diagnostic aids in identifying serious infection and sepsis; however, they do not always show a linear correlation with the risk of organ dysfunction, and despite their elevated sensitivity, they lack specificity [1,6]. Moreover, these inflammatory indexes may also rise in children with viral infections, and although they have been shown to be significantly more elevated in those with underlying bacterial illness, a validated cutoff below which bacterial infection can be conclusively excluded has not yet been established [7].

From this perspective, presepsin (P-SEP) seems to be a promising new marker for the early detection of sepsis. P-SEP (or sCD14-ST) is a fragment of CD14 (cluster of differentiation 14), which is a soluble part of the lipopolysaccharide (LPS) receptor. The LPS receptor is a toll-like receptor that recognizes pathogen-associated molecular patterns (PAMPs) and activates the innate immune response [8,9] which is the organism’s first defense against microbes [9].

P-SEP can be measured in just under 17 min using chemiluminescence enzyme immunoassay technology (PATHFAST presepsin assay) and a very small blood volume (50 μL). Serum levels increase rapidly in the first 2 h from an infectious insult, reaching a peak blood concentration at 3 h, earlier than either CRP or PCT (which peak at around 6 h and 4 h from infection, respectively) [5]. Its half-life of 8 h is also shorter than that of both CRP and PCT [5], so its overall faster kinetics make it potentially more useful for the early detection of severe bacterial infections (Table 1).

Several studies have established cutoffs for P-SEP levels in the blood to diagnose acute bacterial infection or sepsis. In adults, Ghonaim et al. suggested a P-SEP cutoff value of 440 pg/mL for diagnosing sepsis in adult patients, with a sensitivity of 82.5% and a specificity of 90% [10]. In neonates, two studies evaluated cutoff levels for diagnosing neonatal sepsis: the first established a cutoff value of 485 pg/mL, with 97.8% sensitivity and 94.1% specificity [11], and the second established a cutoff value of 538 ng/mL, with 79.5% sensitivity and 87.2% specificity [12]. Furthermore, in a recent study by Pospíšilová et al. [13], P-SEP was evaluated as a potentially effective diagnostic tool for assessing the risk of early-onset neonatal sepsis in newborns.

Overall, as cited in a recent review published by Capossela et al. [5], several authors concur on a critical threshold of approximately 650 ng/L to ensure a sensitivity greater than 90%. However, there is considerable variability in the suggested cutoffs, with significant differences across various age groups, and further studies will be needed to properly determine the values above which P-SEP can be considered a sign of sepsis for each specific pediatric age group (neonates, infants, and adolescents).

In addition to aiding in the clinical decision making in the case of sepsis, the clinical value of P-SEP may also reside in its ability to distinguish between a viral versus a bacterial etiology of febrile illnesses in children presenting to the ED. The ability to rapidly distinguish between these two possible causes of fever represents an undeniable advantage in an emergency setting. Febrile patients identified as having an illness of viral origin can be correctly managed with supportive therapies, thereby mitigating the over-prescription of antibiotics [14,15] and improving timely hospital discharge whilst allowing quick identification of those with more severe conditions that require thorough monitoring and immediate treatment.

In this pilot study, we describe the use of P-SEP in a pediatric emergency department (ED), as well as its diagnostic value in identifying children with serious bacterial infection and differentiating them from those with conditions of viral etiology.

## 2. Materials and Methods

In this descriptive prospective study, we enrolled patients aged 0 to 18 years who presented to the Pediatric ED of the Agostino Gemelli University Hospital in Rome with fever and suspected sepsis from 1 October 2022 to 31 May 2023. Suspected sepsis was assessed according to the definition provided by the International Consensus Conference on Pediatric Sepsis (2005) [1]. A history of antibiotic treatment 48 h prior to admission was a criterion for exclusion.

In adherence to ethical standards, this study received approval from the hospital’s Ethics Committee (ID 4733, date of approval: 20 January 2022), and written informed consent was obtained from all participants’ families before the commencement of data collection.

Family and personal health histories, symptoms, time of onset of fever, physical examination findings, vital parameters, body temperature, and time upon arrival to the ED were all registered.

For each patient, a blood sample was collected for levels of P-SEP, CRP, and PCT, as well as a complete blood count (including total white blood cell [WBC], neutrophil, and lymphocyte counts), in alignment with standard ED clinical practice.

Based upon presenting symptoms and suspected localization of infection, enrolled patients underwent routine microbiological investigations, including blood culture, urine culture, nasopharyngeal swab for viral genome sequencing, or pharyngeal swab for bacterial culture. Additionally, when deemed appropriate based upon severity of patient presentation and the clinical judgment of the ED physician, more invasive procedures, such as lumbar puncture, stool culture, and serological antibody tests, were also performed.

P-SEP blood levels were analyzed via non-competitive chemiluminescent enzyme immunoassay (CLEIA) by the Mitsubishi PATHFAST machine by LSI Medience Corporation, for which a blood sample of 50 μL is required. All clinical and laboratory data were collected in a database.

Patients were later stratified according to presenting illness and CRP values. The illnesses were categorized based upon the main infectious focus: respiratory tract; ear/throat/eyes; genitourinary tract; gastrointestinal tract; nervous system; bone/skin/soft tissues; or sepsis as its own separate category.

Once subdivided in these categories, CRP values were recorded and stratified in agreement with the values proposed by the Consensus Conference Enaspoc31 (European Network for Antibiotic Stewardship at the Point of Care) as follows: <20 mg/L (no probability of bacterial infection), 20–75 mg/L (probability of bacterial infection with necessity of retest after 24 h), and >75 mg/L (very high probability of bacterial infection) [14]. PCT values were recorded in agreement with the reference physiologic values agreed upon in the pediatric setting for diagnosing sepsis: <0.5 ng/mL (negative), between 0.5 ng/mL and 2 ng/mL (probable), and >2 ng/mL (positive) [16].

According to the primary aim of this study, we further separated patients based upon the confirmed viral or bacterial etiologic agent responsible for illness, which was identified via the previously mentioned microbiological investigations. A *t*-test was conducted to analyze the differences between the two groups, and a *p* value of <0.05 was considered significant. We compared means and standard deviations for P-SEP, CRP, PCT, WBC count, and neutrophil count between illnesses of bacterial versus viral origin.

## 3. Results

In our study conducted at the Pediatric ED of the Agostino Gemelli University Hospital between 1 October 2022 and 31 May 2023, a total of 157 patients were enrolled.

Viral agents were found to be the cause of febrile illness in 64 patients (40.6%), whilst a bacterial etiology was identified in 27 patients (17.2%). The remaining 66 patients (42%) were excluded from our study either because an etiologic agent was not detected or because the presence of bacterial and viral co-infection was found upon microbiological testing (Table 2).

Among the 91 cases that received a definitive microbiological diagnosis, viral etiology was identified in 64 patients (70.3%), mainly from nasal swabs performed in those presenting with symptoms of upper airway infection, bronchiolitis, pneumonia, and other illnesses. The main viruses identified were *rhinovirus*, *adenovirus*, *respiratory syncytial virus*, *influenza/parainfluenza viruses*, and *coronaviruses*.

A bacterial etiology was identified in a total of 27 patients (29.7%), 17 males and 10 females, with an average age of 5.8 years (SD ± 6.6). The agents in this category were detected via blood cultures, urine cultures, stool cultures, or pharyngeal swab cultures. The main bacterium identified in urine cultures was *Escherichia coli*, which was sometimes associated with *Klebsiella pneumoniae*, *Enterococcus faecalis*, *Proteus mirabilis*, *Staphylococci*, and *Pseudomonas aeruginosa*. Blood cultures also mainly registered *Escherichia coli*, *Klebsiella pneumoniae*, and *Pseudomonas aeruginosa* (Table 3).

With this data, comparisons between the two groups (viral versus bacterial infection) were made at admission (T0), resulting in a clear difference. We then performed an unpaired *t*-test using the standard deviation and the average values of P-SEP, CRP, PCT, WBC count, and neutrophil count of patients with infections of bacterial versus viral origin.

Average P-SEP values were found to be 309.04 pg/mL (SD ± 273.2) in viral cases and 526.09 pg/mL (SD ± 657) in bacterial cases. Concerning the unpaired *t*-test calculations, P-SEP was found to have a two-tailed *p* value of 0.0398. Average CRP values were 48.54 mg/L (SD ± 61.6) in viral cases and 70.12 mg/L (SD ± 88.8) in bacterial cases. CRP had a two-tailed *p* value of 0.2386. Average PCT values were 3.8 ng/mL (SD ± 10.7) in viral cases and 25.8 ng/mL (SD ± 63.4) in bacterial cases. PCT had a two-tailed *p* value of 0.0399. Average WBC counts were 12.7 × 10^9^/L (SD ± 4.8) in viral cases and 14.45 × 10^9^/L (SD ± 4.8) in bacterial cases. WBC count had a two-tailed *p* value of 0.1880, making it not significant. Average neutrophil percentages were 55.5% (SD ± 18.7) in viral cases and 57.3% (SD ± 19.7) in bacterial cases. Neutrophil count had a two-tailed *p* value of 0.7216, meaning the difference was not statistically significant (Table 4).

Lastly, there were a total of four (2.5%) confirmed septic patients, two males and two females, with an average age of 4.6 years (SD ± 7.9). Two were neonates, one was an infant, and one an adolescent. Upon arrival, values of P-SEP, CRP, and PCT were determined, and samples of blood and urine were also taken for microbiological testing. Since no germ was detected in the cerebrospinal fluid, it was concluded that the infections had not involved the central nervous system.

The average P-SEP value at T0 of the four septic patients was 3328.5 pg/mL (SD ± 1586.6), the average CRP value was 155.12 mg/L (SD ± 165.8), and the average PCT value was 14.08 ng/mL (SD ± 24.0). One patient presented with CRP and PCT values lower than what is considered significant and had a P-SEP value above 2000 mg/mL (Table 5).

Three patients presented with urinary sepsis and had blood and urine cultures that were positive for *Escherichia coli*. The other was a 17-year-old patient who presented to the ED with fever, headache, right eyelid ptosis, vertigo, and non-food-related vomiting. Registered P-SEP was found to be 4673 pg/mL, and, at the same time point, CRP and PCT were respectively 383.8 mg/L and 50 ng/mL. At the end of the diagnostic process, this patient was identified as having had sepsis in the setting of an underlying brain abscess (*Aspergillus niger* was later isolated on a sample of purulent material obtained from surgical drainage).

## 4. Discussion

Fever is one of the most common presenting symptoms in children admitted to the ED [17], and it is often associated with other nonspecific clinical findings, such as tachycardia and tachypnoea [4]. Upper respiratory infections are among the most frequently diagnosed conditions across all pediatric age groups [18], with the highest rates among those younger than 5 years of age, and fever is the prevalent symptom of these conditions. This only partially explains the difficulty in distinguishing the etiology of fever in a child, especially in the context of an ED where hundreds of children are admitted daily, especially during the winter months. Moreover, symptoms associated with viral infection often coincide with those of bacterial disease [18], making the etiological diagnosis more challenging. This can lead to antibiotic prescription errors [19] and incorrect utilization [20] and contribute to inappropriately long discharge rates and hospital overcrowding [21].

In this scenario, P-SEP seems to have a promising role. P-SEP serum levels increase within 2 h, peaking in 3 h from infection. Also, its half-life of 8 h is shorter than that of CPR and PCT [5].

As previously reported by Memar et al., P-SEP levels tend to be considerably higher at T0 than several hours later, while PCT levels are higher at forty-eight hours, and this supports our observation. Other advantages include the small blood volume (50 μL) required for P-SEP measurements and the rapid results [5].

From our data analysis, we found a statistically significant positivity of P-SEP values in patients affected by bacterial pathogens versus those with viral illness, supporting the possible central role of this novel biomarker in a pediatric ED.

Higher values of PCT seem to be correlated with a bacterial origin of infection. PCT is a precursor of calcitonin, produced by thyroid C cells, and is undetectable in healthy patients’ serum. In cases of bacterial infection, proinflammatory cytokines produced by the immunological response stimulate PCT production by cells of different tissues, with consequent increased blood levels at least 6 h after contact with endotoxins [22].

WBC and neutrophil counts did not differ as much as expected in the two groups. In contrast with the results of a retrospective study conducted in 2021, we did not find a relevant difference in the WBC counts among the two populations [7]. Similarly, the neutrophil count was also not significantly different between the two groups; as studied by Naumenko et al., [23] viral infection stimulates neutrophils and triggers neutrophil activation during the initial immune response, and this agrees with the findings of our study.

In the four patients in our study that developed sepsis, all biomarkers were greatly increased, meaning elevated levels correspond to the probable presence of an underlying septic condition at T0.

A recent meta-analysis performed by Yoon et al. [24] concluded that P-SEP showed higher sensitivity and diagnostic accuracy but lower specificity compared to PCT or CRP for detecting sepsis in children. This may represent a limitation of P-SEP as a biomarker of sepsis; however, we did not find a statistically significant correlation between CRP and WBC counts among the groups studied, suggesting that P-SEP may be the determining factor in helping to distinguish between viral and bacterial etiologies in febrile children.

Sepsis still represents one of the major causes of morbidity and mortality in infants worldwide [1], and its rapid evolution makes the management of sepsis challenging for physicians, so its early detection represents one of most studied fields in critical care medicine.

As demonstrated by Ruud G. Nijman et al. in a prospective study conducted in 2020, defining sepsis based only on vital signs and clinical features yields a large proportion of false positive diagnoses.

The definition of pediatric sepsis [1] includes the presence of at least two of four criteria (altered temperature, altered leukocyte count, tachycardia, or abnormal respiratory rate), one of which must necessarily be altered temperature or altered leukocyte count associated with a proven or suspected infection. In compliance with the “Systematic Review and Meta-Analysis by the Pediatric Sepsis Definition Taskforce of 2021”, it is clear that in order to facilitate the identification and treatment of pediatric sepsis, it is necessary to support the diagnosis with markers of inflammation and organ dysfunction [25].

In our study, among the cases of confirmed sepsis (identified by the presence of bacteria in the bloodstream), P-SEP determined upon admission was always above 2000 pg/mL, and is higher when CRP is more than 75 mg/dL, making it a useful diagnostic finding, especially considering the single septic child who presented with elevated P-SEP but CRP and PCT values that were lower than what is considered significant.

PCT, often considered the primary biomarker for sepsis in current clinical practice, demonstrated limited sensitivity and specificity in our study. This index can rise in various non-septic conditions, including fungal infections, surgery, and burns. Therefore, when evaluated alone, PCT should not be deemed entirely reliable as a standalone sepsis biomarker [22].

Our study had several limitations that could impact the generalizability of our findings. Firstly, the limited number of participants restricted our ability to extensively analyze the variability and distribution of P-SEP levels across a broader population. A larger cohort would have facilitated a more detailed stratification of P-SEP values, potentially enhancing our understanding of its utility in differentiating between various risk categories of sepsis, as well as prognosis.

Furthermore, due to the small number of confirmed sepsis cases (*n* = 4), we were unable to determine a reliable cutoff value for P-SEP that could be used for diagnosing sepsis.

Lastly, our study was limited to an acute care setting; therefore, only P-SEP values upon ED admission were collected. Future research should consider monitoring P-SEP trends at different time points during the course of infection to determine whether this biomarker could also serve as a valuable prognostic indicator for sepsis.

## 5. Conclusions

P-SEP seems to be a promising biomarker for differentiating between viral and bacterial etiologies in febrile children presenting with mild and nonspecific signs and symptoms in the pediatric ED. Given its inherent pharmacokinetics and the minimal blood volume required for testing, in the critical care setting, P-SEP holds particular promise because it may potentially expedite the management and treatment of children at risk of sepsis progression.

While acknowledging the limitations of our pilot study, which was characterized by a small patient sample that may not robustly support specific disease groups and their correlations with P-SEP values, it nonetheless demonstrates the potential of P-SEP measurements to allow clinicians to distinguish between bacterial and viral etiologies in febrile children. Our findings consistently show elevated P-SEP values in bacterial illnesses, suggesting the marker’s potential for facilitating this differentiation.

Our results also clearly demonstrated that markedly elevated P-SEP values (>2000 pg/mL) serve as a significant—and perhaps sufficient—indicator of severe sepsis.

The results obtained from our study appear promising, indicating that further exploration of this matter holds potential for early sepsis detection.

## Figures and Tables

**Table 1:** Comparison of sepsis biomarker kinetics after an infectious insult.

Features	P-SEP	CRP	PCT
Initial rise in serum concentration (hours)	2	6–8	2–4
Peak serum concentration time (hours)	3	24–48	24
Half-life (hours)	8	19	22–35

P-SEP, presepsin; CRP, C-reactive protein; PCT, procalcitonin.

**Table 2:** Characteristics of all subjects presenting with febrile illness.

Patient Characteristics	
Total	157
Age, years	4.7 (0–17.8)
Sex, male	73 (46.5%)
Sex, female	84 (53.5%)
Etiological agent of febrile illness	
Unidentified or co-infected	66 (42%)
Identified	91 (58%)
*Virus*	64 (70.3%)
*Bacteria*	27 (17.2%)

**Table 3:** Characteristics of subjects with an identified microorganism responsible for febrile illness.

Patients (*n* = 91)	Viral (*n* = 64)	Bacterial (*n* = 27)
Age, years	2.6 (0–16.2)	5.8 (0–17.9)
Sex, male	35 (54.7%)	17 (63%)
Sex, female	29 (45.3%)	10 (37%)
Final Diagnosis		
Respiratory	Upper airway infection Bronchiolitis Pneumonia Acute asthma	Pneumonia Upper airway infection
Ear, Throat, Eyes	Pharyngitis Conjunctivitis Acute otitis media	Pharyngitis
Genitourinary		Urinary tract infection Pyelonephritis Epididymitis
Gastrointestinal	Acute gastroenteritis	Acute gastroenteritis Appendicitis Appendicular abscess
Neurologic	Febrile seizure Facial nerve palsy	Febrile seizure
Bone, Skin, Soft tissues	Skin rash Transient synovitis of hip IgA vasculitis Kawasaki syndrome	
Microorganisms identified *		
	Nasopharyngeal swab *Rhinovirus* (*n* = 27) *Adenovirus* (*n* = 20) *Respiratory Syncytial Virus* (*n* = 13) *Enterovirus* (*n* = 9) *Coronavirus* (*n* = 6) *Parainfluenza virus types* *1/3* (*n* = 6) *Bocavirus* (*n* = 5) *Metapneumovirus* (*n* = 5) *Influenza type A virus* (*n* = 3) *Influenza type B virus* (*n* = 2) Lumbar puncture *Human Herpesvirus 6* (*n* = 1) Serological antibody testing *Ebstein-Barr Virus* (*n* = 1)	Pharyngeal swab *Aerobic bacteria* (*n* = 1) Blood culture *Escherichia coli* (*n* = 1) *Klebsiella pneumoniae* (*n* = 1) *Pseudomonas aeruginosa* (*n* = 1) *Proteus mirabilis* (*n* = 1) Urine culture *Escherichia coli* (*n* = 13) *Enterococcus faecalis* (*n* = 6) *Klebsiella pneumoniae* (*n* = 3) *Proteus mirabilis* (*n* = 2) *Staphylococci* (*n* = 2) *Pseudomonas aeruginosa* (*n* = 1) *Streptococcus agalactiae* (*n* = 1) *Citrobacter freundii* (*n* = 1) *Klebsiella oxytoca complex* (*n* = 1) Stool culture *Clostridium difficile* (*n* = 1)

* Multiple microorganisms of the same category (viral/bacterial) may be identified in a single patient. Patients with viral and bacterial co-infections were excluded from the study.

**Table 4:** Biomarker analysis—differences in patients with viral and bacterial febrile illness.

Laboratory Tests (Mean, SD)	Viral Illness	Bacterial Illness	*p* Value
P-SEP (pg/mL)	309.04 (SD ± 273.2)	526.09 (SD ± 657)	0.0398
CRP (mg/L)	48.54 (SD ± 61.6)	70.12 (SD ± 88.8)	0.2386
PCT (ng/mL)	3.8 (SD ± 10.7)	25.8 (SD ± 63.4)	0.0399
WBC (×10^9^/L)	12.7 (SD ± 4.8)	14.45 (SD ± 4.8)	0.1880
N (%)	55.5 (SD ± 18.7)	57.3 (SD ± 19.7)	0.7216

P-SEP, presepsin; CRP, C-reactive protein; PCT, procalcitonin; WBC, white blood cell count; N, neutrophil percentage.

**Table 5:** Biomarker analysis—differences in patients with confirmed bacterial sepsis.

Patients with Confirmed Sepsis (*n* = 4)	
Age, years	4.6 (SD ± 7.9)
Sex, male	2 (50%)
Sex, female	2 (50%)
Laboratory Tests (mean, SD)	
P-SEP (pg/mL)	3328.5 (SD ± 1586.6)
CRP (mg/L)	155.12 (SD ± 165.8)
PCT (ng/mL)	14.08 (SD ± 24.0)

P-SEP, presepsin; CRP, C-reactive protein; PCT, procalcitonin.

## Data Availability

Data are contained within the article.
